# Children and Disease*Read before the South Dakota State Medical Society, at Salem, S. D., June, 1892.

**Published:** 1892-09

**Authors:** G. E. Martin

**Affiliations:** Carthage, South Dakota


### CHILDREN AND DISEASE.*

BY G. E. MARTIN, M. D., CARTHAGE, SOUTH DAKOTA.

A subject in which we, as physicians, and especially those who are parents, and also those who expect to be, are deeply concerned about.

We find a great variety of symptoms in diseases of adults, but more especially in children do we have the same disease, showing more variety of symptoms and a greater range of extremes and endurance and inherited tendency to take on or throw off disease.

This is the time that diseased impressions are made upon the life that will show in future years, and gradually, the victorious, (in most cases) sap the vitality of the future man or woman.

The remarkable peculiarities which all diseases assume in children, the results, the rapid changes from one of recovery to danger and *vice versa,* all make this study of children's diseases very important.

At first obscure and difficult of detection, these diseases quickly gain ground and intensity, and run, uncontrolled by any means within our reach. Or the disease may be progressing as well as can be expected, we may have assured the anxious parents that child was improving so far as you could see, when in a few hours you are summoned and arrive in time to see child in convulsions, to be relieved in death. Children endure colds and lung diseases better than indigestion and bowel diseases, showing the great responsibility and necessity of proper and nourishing food at all times.

On account of the nerve centers being very sensitive, they are easily impressed, inducing spasms or laying a foundation for future nerve disease. Here remedies and disease combat on equal grounds. In childhood, nursing and care do more than for adults. Nature is kind and will do more than for adults, showing a wise provision of the Creator in care of the helpless. We have to be on the alert and ever looking out for sudden changes and aggravated symptoms that are prone to occur

*Read before the South Dakota State Medical Society, at Salem, S. D., June, 1892.

and complications that are least looked for. We were sadly impressed with these hidden symptoms of disease in the death of our dear child, in March last, from that dreadful disease, scarlet fever; commencing and running mildly and progressing favorably in all respects, eruption and commencing to scale. Bidding me good-bye on leaving it at four o'clock in the morning, at eleven o'clock, on returning, I saw but too plainly strong symptoms of blood poison

Everything possible was done for him, calling two other physicians in council, but they could only tell what was only too plain. The great responsibility that we, as physicians, should feel in treating diseases of children, none can know but those who as parents have lost a dear child. While the world at large does not hold us as responsible in the treatment of children as adults, we should be held more responsible both financially and morally, because we have the care of one who cannot help itself, and the present and future health and success of the future for child and coming generations. The most responsible trust which we, as physicians, are given.

Here we find no fashionable sickness, no assumed or put-on symptoms. To be sure, we cannot depend on answers to your questions, neither are you mislead by false answers, as is often the case with adults.

You are placed more on your own resources for a correct diagnosis. Yet with close attention and study of all symptoms and expressions, such as the cry, position, countenance, and attentive physical examination, closely studying answers as given by mothers and attendants, we can form our diagnosis almost as correct as with adults.

In the treating of children's diseases, we find that medicine and remedies act with greater range and with less certainty than they do in adults. They have to be given more guarded and watched closer, and with the same diligence we have to watch the disease. In diseases of children, more depends on correct diagnosis than with adults, because often a disease is starting, that if allowed to run or progress, can only with long and persistent efforts be undone, and many are the mistakes that have been made. I will cite a few.

We attended an old lady, 72 years old, with slight cold, the last month, who in her eight years had been blistered for two broken ribs, supposing it was her lungs. We know of a child treated for stomach trouble who had fallen from a load of hay and fractured the skull. My brother's child had been treated by four of the best physicians in Chicago, the past year, for rheumatism, hip joint disease, and tuberculosis, and among them was a specialist. (Here, let me say, is where the specialists make their greatest mistakes by incorrect diagnosis). The child, while jumping the rope, fell with his knee on a stone door sill, and after having knee opened and pus taken out five different times, and knee placed in a cast, is doing well.

Meigs, in Diseases of Children, states that a child does not secrete tears until third or fourth month. We have all attended cases where children have cried good big tears at birth.

I cite these cases to show the necessity of close and correct diagnosis, and that we can go by no rule except close and attentive study of each individual case at the bedside.

In treating diseases of children, that of most importance is to guard well and see that the food is of best and of sufficiently nourishing quality. We cannot attach too much importance to the food, not only in disease, but as a preventive of disease. And at this day and age parents need to be impressed more about the physical care and bringing up their children than the mental training, and brought to see the importance and necessity of a good sound body. The bright, good-looking, always clean child and smart little fellow is often simply living its life; while the slow and often indolent, always in the dirt, is storing up force and makes the active man of the future.

The small symptoms and little events are to be closely watched, for then is the time to prevent and do the most good in children's diseases. The surroundings and different ways of raising children and ways of living, are so various that the proper way to raise a child is a decided mystery, and we are often at a loss to know how to give best advice. For as we go from house to house and see children that have all that can be asked for, clothing, care and food, not stand diseases as well as one that has scarcely food to keep it alive, nor clothing enough to cover it, and poor at that; no fire in house all winter except in the rickety old cook stove or burning hay or chips, with room so cold that we are cold with our wraps on. Yet we have seen these children go through severe cases of eruptive fevers and lung fevers better than the well-cared-for child with no difference in constitution or hereditary taint so far as could be seen. They are the first to be barefoot in spring; in fact a saying is, "you can't kill them." These facts partially show us the condition of children and diseases.

